# Myocardial Fluid Balance and Pathophysiology of Myocardial Edema in Coronary Artery Bypass Grafting

**DOI:** 10.1155/2020/3979630

**Published:** 2020-05-20

**Authors:** Rita Zahara, Anwar Santoso, Adhiala Z. Barano

**Affiliations:** ^1^National Cardiovascular Center Harapan Kita, Jakarta, Indonesia; ^2^Department of Cardiology–Vascular Medicine, Faculty of Medicine, Universitas Indonesia, Depok, Indonesia; ^3^Badau Bergerak Hospital, Kapuas Hulu, West Kalimantan, Indonesia

## Abstract

Myocardial edema is one of the most common complications of coronary artery bypass grafting (CABG) that is linearly related to many coronary artery diseases. Myocardial edema can cause several consequences including systolic dysfunction, diastolic dysfunction, arrhythmia, and cardiac tissue fibrosis that can increase mortality in CABG. Understanding myocardial fluid balance and tissue and systemic fluid regulation is crucial in order to ultimately link how coronary artery bypass grafting can cause myocardial edema in such a setting. The identification of susceptible patients by using imaging modalities is still challenging. Future studies about the technique of imaging modalities, examination protocols, prevention, and treatment of myocardial edema should be carried out, in order to limit myocardial edema occurrence and prevent complications.

## 1. Introduction

Myocardial edema is a medical condition that can be detected in many cardiac conditions [1, 2]. One of the causes of myocardial edema is myocardial injury as occurring in coronary artery bypass grafting (CABG). The increased number of patients undergoing CABG is linear with the increasing number of patients with coronary heart disease (CHD). CHD is the world's leading cause of death, and its incidence is increasing over time in developing countries [3]. The World Health Organization (WHO) reports the number of deaths due to CHD to be 7.2 million per annum or approximately 12% of the total causes of deaths in the world [4, 5]. In Indonesia, by 2002, the reported CHD mortality is reaching 150 per 100.000 population, the highest among Southeast Asian countries, while the reported mortality of CABG in 2014 is 4.95% [6–9].

Myocardial edema is the earliest sign of cardiac dysfunction that later can contribute to post-CABG mortality [10, 11]. A small increase in the amount of myocardial fluid content can cause some significant systolic and diastolic disturbances [12]. Laine and Allen [13] reported that a 3.5% increase in myocardial fluid content could reduce cardiac output as much as 40%. Also, myocardial edema can cause some complications such as arrhythmia and interstitial tissue fibrosis [11, 13, 14]. In order to minimize the occurrence of postoperative myocardial edema, some procedures such as off-pump CABG, isooncotic cardioplegia, and administration of anti-inflammation glucocorticoid have been applied [15]. Unfortunately, little or none of the procedures give satisfactory results in limiting the occurrence of myocardial edema as evaluated by magnetic resonance imaging (MRI), the gold standard for myocardial edema detection [16, 17]. This review will discuss physiological regulation of myocardial fluid balance that contributes to myocardial edema in general and more specific factors in CABG that cause myocardial edema.

## 2. Myocardial Edema

The word edema is derived from the ancient Greek word “*οιδεω*” (means to swell). It is an acute, nonspecific, and reversible condition that can be acquired during cellular injury. In this context, the word myocardial edema refers to cardiac muscle cell swelling or vasogenic (interstitial) swelling in nature [18]. Myocardial edema is defined as a condition where cardiac muscle cells are occupied by an excessive amount of fluid exceeding their physiological level and in turn causing cellular swelling [10]. Myocardial edema is included in one of the essential components in the pathophysiology of ischemic heart disease and nonischemic heart disease such as myocardial infarct, myocarditis, Tako Tsubo cardiomyopathy, and graft rejection [19]. Experts believed that this condition is one of the consequences of CABG and some other cardiac surgeries. Garcia-Dorado et al. [10] mention that myocardial edema following surgical intervention is related to myocardial dysfunction. This condition is frequently mentioned as a secondary process of inflammation and ischemia [12]. Unfortunately, myocardial edema can cause persistent heart dysfunction even though the primary pathological state has already been managed.

Dysfunctions that are caused by myocardial edema include systolic dysfunction, diastolic dysfunction, cardiac arrhythmia, and tissue fibrosis. One of the consequences of myocardial edema is increased in hydrostatic pressure within the interstitial compartment that can exacerbate the extent of necrosis by capillary compression [20, 21]. Myocardial edema also results in decrement of cardiac output, reduction of ventricular compliance, and myocardial stunning. Cardiac arrhythmia and conduction disturbance increase myocardial stiffness and reduce compliance that can affect the systolic and diastolic function and alter the myocardial cellular fluid composition. Myocardial edema is an essential marker in determining the acuity, severity, and extent of tissue injury *in vivo* [1, 13, 22].

In a physiological condition, the interstitial fluid pressure of myocardial tissue range between 15 and 120 mmHg (from end-diastolic to end-systolic phase). Myocardial tissue has a wide cyclical variation of interstitial fluid pressure, but it could not show a negative value during the cardiac cycle [23, 24]. Myocardial end-diastolic interstitial fluid pressure is increased in the case of edema [24]. Capillaries and lymphatic vessels' functions are disrupted during the systolic phase and in turn limit blood flow and transmicrovascular fluid flow from coronary vessels into interstitial space of myocardial tissue. In an increased microvascular permeability setting, edema begins to form in parenchymal tissue of an organ, and the condition alters the cardiac function and modulates the transmicrovascular fluid flow rate or the formation of edema [25].

As mentioned earlier, interstitial myocardial edema is also controlled by the action of lymphatic flow, as this pathway helps the removal of fluid in the interstitial compartment. Edema resolution is affected by the contraction of myocardial tissue itself, the force of contraction, and heart rate. When the myocardial contraction mechanism is crippled, lymphatic flow decreases edema resolution, causing tissue malfunction. Lymphatic vessel of the heart is equipped by a valve that can prevent backflow of fluid, as the heart contraction changes from systolic to diastolic phase over time. [26].

Water occupies 80% of the myocardium, and approximately 78% of the water is in the intracellular compartment [21]. Researchers have suggested that a small increase in the amount of myocardial fluid content can cause some significant systolic disturbances. Foglia et al. [27] mention that an increase of myocardial fluid content by 2.6% after CABG can reduce the left ventricle function by as much as 43%. Laine et al. [13] mention that an increase in myocardial fluid content by 3.5% can reduce cardiac output by 30–50%. Myocardial edema can also cause diastolic dysfunction [13]. The pathophysiology of systolic and diastolic functions in myocardial edema is still unclear. Myocardial edema can also reduce ventricular compliance [28]. The increase of interstitial fluid can increase microvascular resistance, thus reducing coronary perfusion. Increased diffusion distance to myofibril causes a decrease of oxygen diffusion and inadequate oxygen supply. Both conditions can lead to myocardial ischemia. Pathophysiology of myocardial edema is similar to edema of other organs and can be caused by a disturbance in fluid body regulation. Body fluid regulation can be classified into cellular, tissue, and homeostasis of whole-body fluid that will be discussed later [10].

## 3. Myocardial Fluid Regulation

Fluid regulation is essential in order to support an organism's life. Homeostasis of water in cardiac tissue is determined by microcirculation and lymphatic drainage. Cardiac muscle cells can control their cellular volume by modulating water transport across the plasma membrane based on their osmotic gradient and hydrostatic pressure. The myocardial cells can adjust their volume by water transport across the plasma membranes down the osmotic gradient and the hydrostatic pressure [29]. Water moves in a paracellular and transcellular manner by diffusion or through ion channels in the cell membrane and via specific water channels called aquaporins [30, 31].

## 4. Osmotic Force

The water molecule moves through a cell membrane passively. The movement is affected by osmotic gradient and membrane permeability to water. In state of homeostasis, cardiac muscle cells retain their membrane potential by the action of the sodium-potassium pump (Na^+^-K^+^-ATPase). This mechanism carries three sodium molecules from inside the cell to interstitial fluid and two potassium molecules from outside the cell to the intracellular compartment. The action of the sodium-potassium pump depends on the availability of adenosine triphosphate (ATP) as the energy source. In a condition where ATP is limited, the sodium-potassium pump action can cause sodium ion accumulation inside the cell, thus pulling water to the intracellular compartment [10].

Metabolic dysfunction caused by low oxygen supply into cells activates anaerobic metabolism producing lactic acid. Lactic acid accumulation causes metabolic acidosis that should be immediately managed in order to prevent cellular death. In the condition of ischemia, such a process occurs causing an increase of intracellular fluid as sodium accumulates inside the cell. There is an exchange of hydrogen ion and sodium facilitated by Na^+^-H^+^ pump and Na^+^-HCO^−^ cotransporter in order to maintain acidity status. As a consequence, myocardial edema occurs [10].

Usually, the intracellular volume change is temporary. Homeostasis of fluid balance is achieved through volume adjustment and cardiac muscle cell volume adjustment mechanisms. These mechanisms control ion movement through cellular membrane and control organic osmolytes. Organic osmolytes and ions (mainly, potassium and chloride) are released from an intracellular compartment in order to reduce cell volume, and cell shrinkage activates a particular ion transporter that increases organic osmolytes in order to restore the osmotic balance between the two compartments. This mechanism is achieved through activation of Na^+^-K^+^-2Cl^−^ cotransporter pump, as well as the increase of Na^+^ and Cl^−^ ion through Na^+^-H^+^ and Cl^−^-HCO^−^ exchange [10]. Glycogen catabolism and phospholipid membrane also support the fluid movement to the intracellular compartment with the increase of organic osmolytes concentration.

## 5. Membrane Channel

There are three types of transmembrane channels involved in cellular fluid regulation: aquaporin (AQP), connexins (Cx), and pannexins [10]. Aquaporin is a membrane cell protein that forms pores and is highly permeable to water. There are 13 types of AQP in mammals. In cardiomyocytes, there are at least 8 AQPs available. They are AQPs 1, 3, 4, 5, 7, 9, 10, and 11. The most common AQPs that are widely studied are AQPs 1, 3, 4, and 7. AQP-1 is found in capillary blood vessels and lymphatic vessels and contributes to the making of some spaces for water movement in blood vessels, interstitial compartment, and lymphatic vessels. AQP-4 expression is known to increase in ischemia, and the level of expression is also increased as the infarction area becomes increased. AQP-7 is found on the cardiac capillaries and contributes to endothelial cell volume regulation and extracellular edema. This finding shows that aquaporin has a role in ischemia-reperfusion injury through its function in cardiomyocytes volume regulation. Connexin is a protein that forms hemichannel and has a role as a communicator between two adjacent cells [10]. It arranges pathways that will be passed by fluid through the plasma membrane. Gap junction is one of the examples of fluid pathway arrangement by connexin protein. Connexin can manage cellular fluid volume through protein signaling. Pannexin has a slightly similar property as connexin; it forms a transmembrane channel that is important in cell volume regulation.

## 6. Detection of Cell Volume

Mechanical and biochemical processes regulate the detection of cell volume [10]. The mechanical process is facilitated by a channel that has a sensitivity to mechanical changes in the plasma membrane. The channel will be open when the cell membrane is stretched and makes it possible for a water molecule to be freely moving. Sarcolemma has an essential role in cell response to volume changes [10]. Caveolae is a particular part of the cell membrane found in myocytes and endothelial cells. It is an invagination on cell surface approximately 50–100 nm in size, and caveolin-1 is the marker found on caveola [32]. This structure has an important role in transcytosis, both in endothelial cells and epithelial cells. Transcytosis is a process in which macromolecule moves from blood vessel lumen to subendothelial tissue. Transcytosis is affected by three matters; namely, (1) the amount of caveolae on the cell surface can be changed based on its specific signal that can change lipid composition of caveolae, (2) protein caveolin-1 expression level can be increased or decreased, and (3) caveolae receptor location can be changed. Caveola or caveolin-1 can retain vascular permeability. One of the most critical factors that can increase vascular permeability is endothelium-derived nitric oxide (eNOS). Caveolin-1 reaction with eNOS can decrease the activity of eNOS; thus, vascular permeability can be maintained. Decreased expression of caveolin-1 can significantly increase vascular permeability to albumin, thus resulting in tissue edema.

## 7. Intracellular Fluid Distribution

Macromolecule in the cardiac muscle cell is surrounded by a fluid called bound water [10]. The macromolecules are covered by the fluid which acts as a source of hydration. Bound water has a different physical property compared to free water that has a lower freezing point, and it can quickly diffuse compared to free water. Magnetic resonance imaging (MRI) can detect the presence of bound water with the spectroscopy method. In case of cell disruption, bound water will be released from a cell, followed by an increase in intracellular free water without the total cell fluid increasing.

In ventricle muscle cells, mitochondria can manage 40% of the total volume between myofibril and plasma membrane [10]. Mitochondria can tolerate an increase of volume until 30% without altering its integrity. Swelling in mitochondria can occur due to osmotic water movement from the cytosol to the mitochondrial matrix. Changes of osmolality in the cytosol can affect mitochondria volume. Increased volume in the mitochondria matrix can affect the electron transport process and ATP production, reactive oxygen species (ROS) production, apoptosis, and many other cellular processes in mitochondria.

## 8. Tight Junction

One of the endothelium functions serves as a physical barrier between intravascular and perivascular tissues, as well as permeable membrane [33]. Permeability in fluid and molecule transport has two main pathways, transcellular and paracellular. In the transcellular pathway, molecules pass through the apical and basal cells, facilitated by ion channel, protein carrier, an ion pump, and vesicle. In the paracellular pathway, molecules pass through a gap between cells, facilitated by the tight junction. The tight junction is formed by transmembrane protein (occluding, claudin, and junctional adhesion molecule-A) and intracellular protein. Paracellular permeability regulation involves many signaling pathways between the cell and structural components from the cell itself, including tight junction. One of the most studied tight junction regulators is protein kinase-C (PKC). PKC activation can increase tight junction formation, but, on the other hand, it decreases the function of the tight junction as a formed barrier. Another regulator acting on tight junction function is the Rho-associated protein kinase (ROCK). ROCK activation can increase permeability through contraction process using actin and myosin that can cause a partial opening of the tight junction. Other factors that can regulate tight junction integrity are tyrosine kinase, cAMP, and cGMP.

## 9. Fluid Regulation at the Tissue and Systemic Levels

At the organ level, the fluid balance depends on net water filtration in capillary tissue and lymphatic drainage [10]. Capillary filtration depends on intravascular and interstitial hydrostatic pressure, oncotic pressure in two compartments, microvascular filtration coefficient (depending on microvascular surface area and permeability to fluid), and Staverman osmotic reflection coefficient. Those components are included in Starling-Landis modified equation. Net capillary filtration is the total result of filtration in a capillary vessel. Capillary filtration shows the negative result at the distal part or in pathological condition.

Lymphatic drainage is managed by the cardiac pump cycle and depends on central venous pressure [10]. Any condition which increases capillary filtration or decreases lymphatic drainage will increase fluid content in the myocardium. Capillary filtration will be increased in case of increased hydrostatic pressure, decreased intravascular oncotic pressure or increased interstitial oncotic pressure, and an increase of microvascular filtration coefficient.

## 10. Myocardial Edema in CABG

CABG can be carried out via two methods: on-pump or off-pump method. On-pump CABG is carried out using a cardiopulmonary bypass (CPB) machine [15]. In CABG, the heart is crippled during the procedure, and the use of CPB machine can assist the cardiovascular surgeon in maintaining circulation. The CBP machine is an essential extracorporeal circulation that functions as a heart during CABG. The use of the CPB machine can cause some complications to the heart, lung, kidney, and brain [15]. The mechanisms behind those complications are multifactorial, and one of them is systemic inflammatory system activation, commonly called systemic inflammatory response syndrome (SIRS). Some contributing factors to SIRS development include (1) contact between plasma and cannula surface (identified by patients' body as foreign object), (2) shear forces between plasma and CPB machine, (3) hypothermia, and (4) ischemia-reperfusion injury. In SIRS, some inflammatory components are released, such as TNF, IL-6, IL-8, C3a complement, 5a complement, neutrophil, leukocyte, and monocyte [15, 34]. In the same time, there is an activation of coagulation followed by thrombus and fibrinolysis. There is an increase in capillary permeability, white blood cell activation, and thrombus formation that, in turn, can cause damage to endothelial of blood vessels followed by organ dysfunction. Many alternatives have been applied in order to minimize SIRS in CABG, which are (1) off-pump CABG technique, (2) heparin-bond circuits, (3) leucodepletion filter, and (4) pharmacological modification with glucocorticoid, complement inhibitor, and aprotinin [15]. Despite attempts to reduce SIRS in on-pump CABG, the occurrence of SIRS is inevitable.

Other methods of CABG are called off-pump CABG and minimally invasive direct coronary artery bypass (MIDCAB) [15]. These procedures are still in development, in order to reduce systemic complication of CABG and reduce post-CABG morbidity and mortality. However, various meta-analyses comparing on-pump and off-pump CABG have failed to demonstrate such expectation by using newly developed methods. Meta-analyses by Reston et al. [35] indicate reduction in the incidence of myocardial infarct, stroke, bleeding, renal dysfunction, atrial fibrillation, and infection of operation wound in off-pump technique, even though, in the long-term follow-up, mortality and redo-revascularization are lower in on-pump CABG.

There are two main differences between on-pump and off-pump CABG. In off-pump CABG, a CPB machine is officially unused, and the ischemic-reperfusion injury is minimal [36]. Unfortunately, tissue trauma, heart manipulation, suction pericardial, and administration of many pharmacological agents, such as heparin and protamine, are still occurring and used [37]. In the early phase of CABG, inflammatory factors (e.g., TNF-*α*, IL-8, IL-10, and complement) increase and they are of higher levels than the levels present in on-pump CABG. After the procedure, there are no differences between the two. Castelheim [38] researched 25 inflammatory biomarkers involved in CABG. Only a complement system is significantly activated in on-pump CABG, while an increase of other inflammatory biomarkers is not significant in the two procedures.

In cardiac arrest, there is a decrease in lymphatic drainage that can contribute to myocardial edema [10, 26]. While the CPB machine is working, the heart is arrested in diastole, thus causing some events such as (i) contraction problem, which in turn causes lymphatic drainage to be crippled; (ii) a long diastole duration during procedure causing increase in transmicrovascular fluid flow [39]; (iii) activation of humoral and cellular mediators, increasing microvascular permeability and thus microvascular fluid filtration [40–42]; (iv) colloid osmotic pressure reaching nearly zero due to crystalloid solution used to arrest cardiac organ and perfuse coronary vessel [43]. Lack of cardioplegic fluid colloid can also cause loss of fluid from cardiac vasculature, as we know that one of the factors that cause intravascular fluid to stay inside the vessel is colloid osmotic pressure determined by plasma protein albumin, although additional experimental data have not been reported [44].

During cardioplegic agent administration, the myocardium is in ischemia and reperfusion states. In that condition, myocardial edema is limited by cardioplegic agent composition and temperature, because hypothermia can alter cellular metabolism and ion channel activation.

## 11. Ischemia-Reperfusion Injury

Ischemia-reperfusion injury (I/R injury) is defined as the cellular alteration of the previously viable cell after reperfusion [45]. I/R injury is the most important cause of morbidity and mortality after CABG. In CABG, there is a temporary occlusion in the coronary artery. The circulation resumes with a higher quantity of blood compared to preoperative coronary blood flow. There are some underlying mechanisms in I/R injury after CABG, which are apoptosis due to free radical accumulation through anaerobic metabolism during ischemia and intracellular Ca^2+^ overload. Other factors contribute to I/R injury, such as complement activation, leukocyte adhesion to endothelium, aggregation of platelets and leucocytes, increased microvascular permeability, and decrease in endothelial compliance.

Generally, there are four manifestations of I/R injury: (1) *myocyte lethal reperfusion injury*—in this condition, there is necrosis of cardiac muscle cells that were previously viable; histopathologically, the necrosis is called contraction band necrosis; (2) *vascular reperfusion injury*—in this condition, there is progressive microvascular damage that can cause no-reflow phenomena; (3) *stunned myocardium*—in this condition there is myocyte dysfunction that is prolonged due to abnormal intracellular metabolism; (4) *reperfusion arrhythmia*—there is a nonsustained ventricular tachycardia or ventricle fibrillation right after reperfusion procedure [46].

Myocardial condition after I/R injury is marked by myocardial edema [47]. Myocardial edema is identified after reperfusion and occurs regularly one week after the procedure. Edema after I/R injury is not a constant condition in terms of its characteristics, and it somewhat shows a more bimodal pattern as edema that occurs on the postoperation day followed by improvement of edema afterwards and then the reoccurrence of edema days after reperfusion and resumes until one week after reperfusion. The mechanism of the bimodal pattern is still unknown. Many researchers mention that the first edema reaction is caused by reperfusion itself, and the second reaction is caused by the tissue healing process.

## 12. Identification of Myocardial Edema

Cardiac imaging studies have been advancing, especially for echocardiography, computed tomography scan (CT scan), and magnetic resonance imaging (MRI). Echocardiography still becomes one of the most frequently used modality in cardiology despite its two-dimensional image. It is still popular due to its availability, being a more effortless operation, economic reasons, and real-time imaging [48]. Detection of myocardial edema with this modality relies on left ventricle mass and wall volume measurement. Both measurements increase in the presence of myocardial edema, even though the increase of ventricular mass may not be specific for myocardial edema. Dent et al. [49] reported the ability of high-frequency ultrasonic imaging to characterize myocardial edema presence.

In the study, the myocardial water content can be characterized by the technique, allowing quantification of changes in myocardial properties. Detection of myocardial edema is demonstrated by Powell et al. [50]. The modality has been useful in assessing vascular function and anatomy of the organ. Unfortunately, inferior temporal and spatial resolution and reported motion artifacts have limited CT scan to quantify myocardial edema in a clinical setting. Mahnken et al. [51] reported a dual-source CT scan ability in detecting myocardial edema when used with other modality in a porcine acute myocardial infarct model. MRI has demonstrated to serve as a tool that can evaluate the function and anatomy of cardiac tissue. Despite its superiority in detecting heart function and anatomy, the high cost and availability limit its usage. Kiricuta and Simplăceanu [52] reported a correlation between tissue hydration and T1 and T2 (longitudinal and transverse) relaxation times that became the benchmark of myocardial edema detection using MRI as it helps in quantifying changes in myocardial water content. Higgins et al. [53] mention the linear relationship between myocardial water content and T2 relaxation time in the acute myocardial infarct. Transmural distribution of myocardial edema is demonstrated by Karolle et al. [54] using T1 and T2 relaxation times that are lengthened after myocardial ischemia. Albers et al. [55] show the feasibility of T1-weighted MRI to detect myocardial edema induced by crystalloid cardioplegia usage. Nonuniform three-dimension distribution of myocardial edema is demonstrated in the study. T2-weighted MRI has been used to examine myocardial edema and also the area at risk associated with acute ischemic injury of cardiac tissue. When T2-weighted MRI is used with other modality, it can be used to detect myocardial ischemia related to myocardial edema before irreversible injury [56]. With recent studies and findings, MRI proves to be one of the most promising modalities in the detection of myocardial edema that can help clinicians to diagnose and determine the prognosis when used together with other modalities. More specific MRI algorithm is being evaluated to be focused on detecting myocardial fluid content and overcome limitations such as low signal-to-noise ratio, motion artifacts, and many other limitations, especially in T2-weighted technique [57, 58].

## 13. Challenges and Recent Development

Identification for susceptible patients with myocardial edema is crucial. Providing availability of diagnostic modalities, such as echocardiography, CT scan, and MRI, is one of the critical challenges. Recent studies show the benefit of using MRI as a diagnostic tool for myocardial edema detection. However, the availability and information about the technique of identification are still limited. MRI examination protocols should be provided for more consistent image quality. The development of T2 mapping may contribute to a better imaging of myocardial edema. Visual interpretation is mostly subjective. Thus, studies regarding T2 mapping should be further investigated in order to provide a universal protocol for the detection of myocardial edema [59]. Information regarding the safety of procedure and cost efficiency should be widely provided. Combined MRI identification of ventricle function, myocardial edema, and myocardial healing process in the form of scar tissue should be considered in patients suspected to have myocardial injury. All modalities examination protocol should be provided in order to produce similar output and identification techniques [57, 59, 60]. Medical management and prevention should be directed to limit the occurrence of myocardial edema and to prevent further damage to cardiac tissue.

In the last two decades, there are improvements in identification, therapy, and interventional procedure in cardiovascular medicine that help cardiologist fight against cardiovascular diseases [61]. Most changes that are implemented on guidelines are mainly due to randomized control trials that are carried out in a large scale. While the prognosis is getting better, many physicians think that a more personalized approach to the management of cardiovascular disease should be addressed because most of randomized studies have some limitations on selection and phenotype criteria and have obviously given the individual character of a single patient and the unique states of disease less consideration. Thus, it is important to characterize the disease based on the patients so that the management can also be personalized [61, 62].

Computational cardiology helps the biologist and clinician to better understand the fundamentals of cardiology and to apply them into clinical application. Some identified roles and challenges of computational cardiology include the identification of new biomarkers without ambiguous information on diseases, the prediction of outcome (intra- and postprocedural) for current and developed pharmacotherapies and interventional cardiology, a real-time approach for integration of data in the hospital and a bigger scale for instance at population level, early identification of health hazards in order to prevent disease, supporting data to health economic approach in high-burden cardiovascular disease, improvement of phenotyping point of care and decentralized care to relieve the burden of hospital setting, introduction to novel or personal drug targets (e.g., possible future application to genetic repair), and an automated monitoring to newly introduced treatment for the early recognition of treatment success and side effects [61]. The innovation of computational modeling targeted precise medicine demands through a simulation of molecular pathways, cells, tissues, organs, and whole organisms' studies [61]. In the practice, molecular and clinical phenotyping will be the fundamental in conjunction with developments in bioinformation method for integration of multilevel high-throughput and high-content datasets with clinical data. It will open a path to integrative approaches to personalized medicine [61]. Today, the utility of mathematical models remains largely removed from clinical setting despite its role in providing knowledge and insights that are not supplied by current data modalities and making evidence generation more efficient by reducing the number of real patients needed in clinical studies.

As a recall to our topics, despite the fact that it is invasive and has a significant sampling error, endomyocardial biopsy is considered the gold standard for detecting myocardial inflammation, a condition that can lead to myocardial edema. New diagnostic tools have been studied to limit the invasiveness and increase diagnostic accuracy. Cardiac magnetic resonance (CMR) provides noninvasive assessment of cardiac function, anatomy, and tissue characterization. New techniques have been developed rapidly, and T2 parametric mapping allows detection of myocardial edema by identifying increased water content at the edematous area with greater accuracy [63]. The introduction of computational modeling can aid the previous modalities to better illustrate the characteristics of a patient's cardiovascular status. Several studies that include computational cardiology have been published. A study by Reis et al. [63] demonstrates a personalized computational model of edema formation in myocarditis based on long-axis biventricular magnetic resonance imaging. In the study, the complex physiological process was mathematically modeled using a nonlinear system of partial differential equations (PDE) based on porous media approach. A new hydromechanical model for inflammatory edema was developed by combining a Biot's poroelasticity theory model and an immune response model. Verification of novel computational model was carried out using T2 parametric mapping obtained by MRI in order to identify the edematous area in patients diagnosed with nonspecific myocarditis. A patient-specific geometrical model was generated using MRI from myocarditis patients. Edema formation was illustrated using the proposed hydromechanical mathematical model in two-dimensional domain. The computer simulations allowed us to correlate spatiotemporal dynamics of representative cells of the immune system (e.g., leucocytes, pathogen, etc.) with fluid accumulation and cardiac tissue deformation. The study shows that the mathematical model is a very promising tool in terms of understanding formation of edema in case of myocarditis. Simulations obtained from patient-specific model reproduced important aspects related to formation of cardiac edema, area involved, position, shape, and how these features relate to immune response [63]. The study implies that a similar technique can be adapted to reconstruct how cardiac muscle cells formed in CABG setting. Another study by Pontecorboli et al. [64] reveals a morphological feature of Tako Tsubo cardiomyopathy using a computational model. The theory said that transient myocardial dysfunction could be identified at the left ventricular apex. The study investigated left ventricle morphology and deformation in 28 patients. Patients with CMR investigation within five days prior to admission showed a reduced left ventricular ejection fraction and a higher ventricular volume, but no differences in electrocardiography, global strains, or myocardial edema. Statistical shape modeling was generated into three modes and was introduced as left ventricle size model, apical sphericity model, and height model. The models suggest that significant differences can be observed in mode 1, left ventricle size model, which suggests that early Tako Tsubo cardiomyopathy left ventricle remodeling is mainly influenced by a change in ventricular size rather than apical sphericity [64]. A study by Lopez-Perez et al. [65] demonstrates a three-dimension cardiac computational model. The study shares the idea of how the advancement of medical imaging over the last decades allows the evolution from conventional to patient-specific three-dimensional computation model. The study analyzes sixty representative three-dimensional cardiac computation models developed and published during the last fifty years, describing their information sources, features, development method, and online availability, and it even includes the necessary components to build those models. The study mentions thoroughly steps on how to craft a personalized three-dimensional cardiovascular model and show a potential usefulness of the model to clinical environment as a tool to assist physicians and clinicians in prevention, diagnosis, and treatment of cardiovascular disease [65]. A study by Zhang et al. [66] investigates the characteristics of flow and distributions of the hemodynamic parameters in a coronary artery bypass model which includes proximal and distal anastomoses under physiological flow conditions. The study identifies disturbed flows in the form of flow separation or reattachment, vortical and secondary flows, and areas of high oscillatory shear index with low wall shear stress and low-oscillatory shear index with high wall shear stress. They were found both in the proximal and distal anastomoses, especially at the toe and heel areas of distal anastomosis. The findings indicate highly suspected sites for the onset of the atherosclerotic lesions. The flow patterns found in the graft and distal anastomoses of our model at deceleration phases are distinctive from those of the isolated distal anastomosis model. As an additional piece of information, a significant difference in segmental averages of hemodynamic parameter was found between proximal and distal anastomoses. The findings suggest that intimal hyperplasia would be more prone to form in the distal anastomosis than in the proximal anastomosis, specifically along the suture line at the toe and heel of distal anastomosis. However, the study includes some assumptions about its symmetrical designed model and ignorance to compliance effect of vessel walls. The blood flow was assumed to be laminar with Newtonian fluid, and the flow of the graft is a scale-down version of the aortic flow waveform [66]. The study may be regarded as the first identification of the flow characteristics and hemodynamic parameter distributions for CABG model, from the aorta to the occluded coronary artery. The flow patterns in distal anastomosis and graft have revealed the differences between present results and previous investigations involving only the distal anastomoses. The results have indicated the potential locations of atherosclerosis lesion formation, which is in line with the in vivo observations. The findings have shown that the investigation of segmental average of hemodynamic parameter is useful and beneficial for quantitatively correlating the hemodynamic studies with clinical features [66]. At last, Guerciotti et al. [67] performed a parametric computation of fluid dynamics in patient-specific geometries with the aim of investigating a possible relationship between coronary artery stenosis degree and risk of graft failure. The study was driven by the fact that CABG may fail in the future due to restenosis, despite an excellent report on patency rates. The findings of the study suggest that low degrees of coronary artery stenosis produce more disturbed fluid dynamics in the graft, resulting in hemodynamic conditions that may promote a higher risk of graft failure [67]. From clinical view, the results confirm the evidence that the long-term patency of left internal mammary artery graft may be greatly affected by the stenosis degree of the native left anterior descending artery, as low degrees of left anterior descending artery stenosis may result in higher hemodynamic indices related to the risk of restenosis in the anastomosis [67]. Limitations of the study are the assumption of rigid walls and a clinical validation of the proposed extended Murray's law that is needed to understand that its real applicability has not been discussed in the study [67].

Previous studies have proven that mathematical or computational models are increasingly popular in biomedical science and applied science. Although they are a simplification of reality, computational models are able to connect multiple processes to each other [68]. It can handle the complex dataset quite well, thus improving diagnosis and healthcare [68, 69]. Previous studies also suggest that cardiovascular system has acquired some advantages from the reductionism (perspective of an organ, that is, break down into tissue, cells, and molecules) and biological integration of the components and their interactions necessary to reveal organ phenotype and function [62].

Specific for device development purpose, the major computational model and technological challenges have been addressed and the authorities and the regulator immediately recognized the benefit. Continuous investment will enable engineers to resume model refinement and develop novel applications, targeting increases in personalization, precision, and speed. In the development, these relatively new approaches should be compared to current practice in terms of some parameters so that the risk and benefit can be measured. Beyond this, efficacy must be demonstrated in large multicenter clinical trials. It is legitimate that these techniques have the potential to change the approach to clinical practice. The future benefit will be in favor of patients, physicians, clinicians, and healthcare providers [70]. Last but not least, the reality forces us to acknowledge that computational modeling is still on a long way from perfection. Every step of collecting and synthesizing data, processing, and generating output is still under development with a close supervision from experts [68].
